# Systematic review and meta-analysis of the efficacy and safety of electroacupuncture for poststroke dysphagia

**DOI:** 10.3389/fneur.2023.1270624

**Published:** 2023-12-06

**Authors:** Xuezheng Li, Lijun Lu, Xuefeng Fu, Hao Li, Wen Yang, Hua Guo, Kaifeng Guo, Zhen Huang

**Affiliations:** ^1^Postgraduate Cultivation Base of Guangzhou University of Chinese Medicine, Panyu Central Hospital, Guangzhou, Guangdong, China; ^2^Department of Rehabilitation Medicine, Guangzhou Panyu Central Hospital, Guangzhou Guangdong, China

**Keywords:** stroke, dysphagia, electroacupuncture, meta-analysis, systematic review, randomized controlled trials

## Abstract

**Introduction:**

Optimal treatment strategies for post-stroke dysphagia (PSD) remain to be explored. Electroacupuncture (EA) has attracted widespread attention due to its simplicity, cheapness, and safety. However, the efficacy of EA in the treatment of PSD lacks high-level evidence-based medical support. This study aimed to systematically evaluate the clinical value of EA in the treatment of PSD.

**Methods:**

A total of seven databases were searched for relevant literature. All randomized controlled trials (RCTs) on EA alone or EA combined with other interventions for the treatment of PSD were assessed using the modified Jadad scale. The studies with a score of ≥4 were included. The quality of the included studies was then assessed using the Cochrane Collaboration’s tool. The meta-analysis was performed using Rev. Man 5.3 software.

**Results:**

Twelve studies involving 1,358 patients were included in the meta-analysis. Meta-analysis results showed that the EA group was superior to the control group in terms of clinical response rate (OR = 2.63, 95% CI = 1.97 to 3.53) and videofluoroscopic swallowing study (VFSS) score (MD = 0.73, 95% CI = 0.29 to 1.16). There was no significant difference between the two groups in the standardized swallowing assessment (SSA) score (MD = -3.11, 95% CI = -6.45 to 0.23), Rosenbek penetration-aspiration scale (PAS) score (MD = -0.68, 95% CI = -2.78 to 1.41), Swallowing Quality of Life (SWAL-QOL) score (MD = 13.24, 95% CI = -7.74 to 34.21), or incidence of adverse events (OR = 1.58, 95% CI = 0.73 to 3.38).

**Conclusion:**

This study shows that EA combined with conventional treatment or other interventions can significantly improve the clinical response rate and VFSS score in patients with PSD without increasing adverse reactions.

**Systematic review registration**: https://www.crd.york.ac.uk/PROSPERO/display_record.php?RecordID=396840.

## 1 Introduction

Swallowing disorders are one of the common complications after stroke, with a prevalence of approximately 37 to 78% in stroke patients (1). Patients with swallowing disorders may suffer from slow food intake, choking on water, and dysphagia, which seriously affect their quality of life (1). Due to a decline in swallowing function, patients are more prone to developing aspiration pneumonia (OR = 4.08, 95% CI = 2.13–7.79) (2) and malnutrition (OR = 0.91, 95% CI = 0.83–0.99) (3) or even death (OR = 4.07, 95% CI = 2.17–7.63) (2). In addition, PSD has also brought a huge economic burden to patients, families, and society. In the United States, the mean hospital stay of patients with dysphagia is 3.8 days longer than that of patients without dysphagia, which will incur an extra mean hospitalization expense of USD 6,243 (4). Therefore, it is necessary to find an effective way to treat dysphagia.

Currently, there are many clinical methods to treat dysphagia, such as swallowing training (5), neuromuscular electrical stimulation (6), non-invasive transcranial direct current stimulation (7), and balloon dilation therapy (8). Although these treatments have been employed to treat dysphagia (9), the optimal treatment protocols for dysphagia remain to be explored (10). It is generally believed that dysphagia in stroke patients is induced by a focal lesion that interrupts the connection between the nucleus tractus solitarius (NTS) and nucleus ambiguus (NA) on the same side of the lesion (11). Studies have revealed that electroacupuncture (EA) is a potential intervention strategy for dysphagia, which can improve the swallowing function through the neural circuit of the “primary motor cortex (M1) - parabrachial nucleus (PBN) - NTS” (12). However, the application of EA in the treatment of poststroke dysphagia (PSD) is still controversial. Some studies have shown that acupuncture, including EA, helps patients recover from post-stroke sequelae, like dysphagia (13, 14). However, other studies have shown that acupuncture has no effect on the recovery of patients’ functions after stroke (15, 16). A previous meta-analysis (17) only included articles comparing the efficacy of EA combined with swallowing training and single swallowing training, and the authors pointed out that the quality of the included literature was relatively poor. Therefore, these findings are inconclusive. More clinical studies on EA for the treatment of PSD have emerged afterward and shown positive therapeutic outcomes.

Therefore, this study included randomized controlled trials (RCTs) on both EA alone and EA combined with conventional interventions. Relevant articles were screened for inclusion or exclusion using the modified Jadad scale. The aim of this study was to conduct a meta-analysis of RCTs on EA and EA combined with other interventions for the treatment of poststroke dysphagia (PSD), in order to provide higher quality evidence-based basis for the clinical rehabilitation of patients with PSD.

## 2 Materials and methods

### 2.1 Study registration

This systematic review protocol was registered on the PROSPERO registration platform (ID: CRD42023396840) on 17 February 2023 and was conducted according to the PRISMA statement (18).

### 2.2 Search strategy

A total of seven databases were searched, including four English databases: PubMed, Embase, the Web of Science, and Cochrane Library; and three Chinese databases: CNKI, Wanfang Data, and China Biology Medicine (CBM). All publications, regardless of country, language, or article type, were searched from the inception of databases to 1 February 2023. A comprehensive search was conducted with stroke, dysphagia, and EA as keywords. Chinese search terms included “stroke,” “apoplexy,” “cerebrovascular disease,” “cerebrovascular accident”; “swallowing,” “dysphagia,” “deglutition difficulty”; “EA,” and “EA therapy.” English search terms included: “stroke,” “acute cerebrovascular lesion,” “cerebrovascular accident,” “CVAs,” “dysphagia,” “deglutition difficulty “, “deglutition disorder,” “swallowing difficult,” “electroacupuncture,” “electro-acupuncture,” and “electronic acupuncture.” The search terms and search formulas were modified and refined according to the requirements of different databases to ensure that all databases were adequately searched. The detailed search strategy is provided in Supplementary File S1.

### 2.3 Inclusion and exclusion criteria

#### 2.3.1 Study type

Firstly, all RCTs on EA for the treatment of PSD were searched, whereas non-randomized studies, observational studies, experimental animal studies, qualitative studies, case reports, empirical summaries, and correspondence were excluded. Secondly, the methodological quality of the literature was assessed using the modified Jadad scale and criteria (19). Based on the scale, the studies achieving a Jadad score of less than 4 were excluded, and all RCTs on EA for the treatment of PSD with a score of 4 or higher were included, which ensured the quality of articles included in this meta-analysis.

#### 2.3.2 Type of participants

All patients met the clear clinical diagnostic criteria for PSD: (1) diagnosis of ischemic or hemorrhagic stroke by CT or MRI and (2) diagnosis of dysphagia by clinical bedside swallowing assessment, videofluoroscopic swallowing study (VFSS), or fiberoptic endoscopic evaluation of swallowing (FEES).

#### 2.3.3 Types of interventions

The experimental interventions of the studies included in this paper included EA alone or EA combined with other interventions such as medication, rehabilitation training, neuromuscular electrical stimulation, and ice stimulation. Except for EA, the rest of the interventions should remain the same between the experimental and control groups (except for conventional acupuncture, sham acupuncture, and other blinded strategies).

#### 2.3.4 Types of outcome indicators

To evaluate the effect of treatment on PSD, we used the following outcomes as primary outcome indicators: (1) clinical response rate, (2) VFSS score, (3) standardized swallowing assessment (SSA) score, (4) Rosenbek penetration-aspiration scale (PAS) score, and (5) Swallowing Quality of Life (SWAL-QOL) score. Secondary outcome indicators were the number and severity of adverse events to evaluate the safety of treatment.

### 2.4 Data extraction

After the search was completed, the retrieved documents were imported into Endnote 20 software. After automatic duplicate removal, the titles and abstracts of the articles were read independently by two reviewers (Li Xuezheng and Guo Hua) to exclude studies that clearly did not meet the inclusion criteria. The full texts of the remaining studies were re-screened to judge whether the articles met the inclusion criteria. Two independent investigators extracted data from the included studies. The extracted data included general information, such as first author, year of publication, sample size, randomization method, and grouping; participant information, such as sex, age, and duration of disease; intervention information, such as the number of interventions, acupuncture points, and EA parameters; outcome indicator data; follow-up outcomes and duration; and adverse events. In case of disagreement, a third reviewer (Li Hao) was consulted for adjudication.

### 2.5 Quality assessment

First, two reviewers (Li Xuezheng and Guo Hua) independently evaluated the methodological quality of the included literature using the modified Jadad scale and criteria (19). Studies achieving a score of 4 or higher were considered high-quality, while those scoring less than 4 were considered low-quality. In case of disagreement, the decision was referred to a third reviewer (Li Hao).

The risk of bias in the studies achieving a score of 4 or higher was then assessed using the Cochrane Collaboration’s tool (20). Items in the tool are divided into seven sectors: (A) Random sequence generation; (B) Allocation concealment; (C) Blinding of participants and personnel; (D) Blinding of outcome assessment; (E) Incomplete outcome data; (F) Selective reporting; and (G) Other bias. The risk of bias in the searched original articles was assessed, and the included studies were rated as having a “Low,” “High,” or “Unclear” risk of bias. Based on the above seven sectors of items, these articles were classified as “Yes” (low bias for A - E; high bias for F - G), “No” (high bias for A - E; low bias for F - G), and “Unclear” (lack of relevant information or presence of uncertain bias). Two reviewers (Li Xuezheng and Fu Xuefeng) independently conducted the assessment. In case of disagreement, a third reviewer (Lu Lijun) was consulted for adjudication.

Finally, the level of evidence for each outcome indicator was assessed independently by two investigators (Lijun Lu and Xuefeng Fu) using the Grade Pro tool from five aspects: “Risk of bias,” “Inconsistency,” “Indirectness,” “Imprecision,” and “Publication bias.” If there was a disagreement, it was resolved by the decision of the third reviewer (Xuezhen Li).

### 2.6 Statistical analysis

Statistical analysis of the included RCT studies was performed using Review Manager 5.3 software. Data included dichotomous and continuous variables. Odds ratios (ORs) with 95% confidence intervals (CIs) were used to represent dichotomous variables; while mean differences (MDs) with 95% CIs were used to represent continuous variables. Heterogeneity among studies was determined by the I^2^ test. If I^2^ < 50%, a fixed-effects model was used for data analysis; conversely, if I^2^ ≥ 50%, a random-effects model was adopted. A funnel plot was used to test publication bias.

## 3 Results

### 3.1 Literature screening process and results

**Figure 1:**
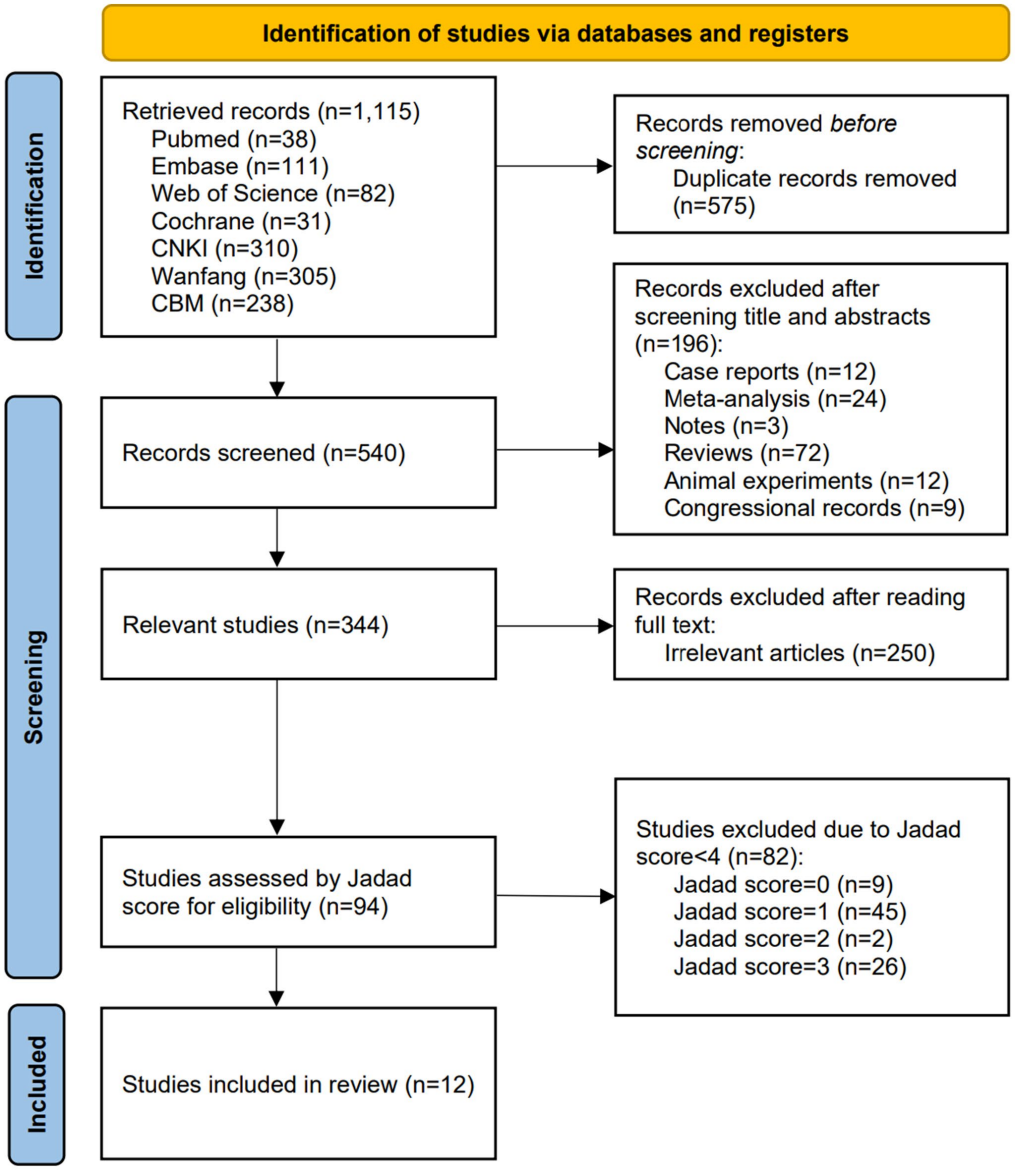
Flow diagram for the selection of the included studies.

The literature screening PRISMA flow chart is shown in Figure 1. Literature was carefully screened for eligibility according to the principles of Population, Intervention, Comparison, Outcomes, and Study (PICOS). The search in seven databases identified a total of 1,115 documents. After excluding 575 duplicates, 540 publications were left. After the initial screening of titles and abstracts, 344 articles were obtained, and 250 irrelevant articles were further excluded by reading the full text. Quality assessment was performed on the remaining 94 articles, and 82 low-quality articles (Jadad score < 4) were excluded. Finally, 12 high-quality eligible articles were included (14, 21–31).

### 3.2 Basic information of the included literature

The basic information of the included studies is shown in Table 1. A total of 12 articles were included, with all the studies conducted in China. They were published between 2011 and 2022, with two of them published in English and the remaining 10 in Chinese. The total number of eligible cases was 1,358, including 747 cases in the experimental group and 611 cases in the control group. In all of these trials, the frequency of EA interventions was at least five times per week and the duration was at least 2 weeks. Two articles (22, 28) included two pairs of trials, five (14, 23, 26, 28, 29) had subject dropout, and three (14, 28, 31) reported subject follow-up, with no follow-up lasting longer than 3 months. The articles reported the following outcome indicators: (1) 10 articles reported clinical response rate (14, 21–23, 25–29, 31); (2) two articles reported VFSS scores (14, 22); (3) five articles reported SSA scores (21, 27–29, 31); (4) three articles reported PAS scores (24, 27, 30); (5) three articles reported SWAL-QOL scores (28, 29, 31); and (6) seven articles mentioned adverse effects (14, 21–24, 28, 29).

**Table 1:** Characteristics of the included studies.

Study	Randomization	Patients (male)		Year (mean ± sd)		Intervention		Treatment duration	Electrical acupoints	EA parameter
		*IG*	*CG*	*IG*	*CG*	*IG*	*CG*			
Chen 2016	RML, CE	125 (74)	125 (74)	62.52 ± 10.60	64.06 ± 10.54	EA + ST	ST	siw, 3 W	GB20, EX-HN14, BL10, GV16, Gongxue, CV23	2HZ, 30 min
Zhang 2011	RML, CE	193 (104)	90 (90)	61.11 ± 6.49	62.2 ± 8.50	EA + RDT	CA + RDT	bid, 30D	EX-HN24, CV23	50HZ, 2 V, 0.2 ms, 30 min
Wang (a) 2014	SPSS, CE	41 (NR)	40 (NR)	NR	NR	EA + ST + RDT	ST + RDT	qd, 3 W	GB20, CV23, GV15	DW, 30 min
Wang (b) 2014	SPSS, CE	41 (NR)	42 (NR)	NR	NR	EA + NES + ST + RDT	NES + ST + RDT	qd, 3 W	GB20, CV23, GV15	DW, 30 min
He 2018	RML, CE	35 (19)	35 (17)	64 ± 6	69 ± 7	EA + ST + RDT	ST + RDT	fiw, 4 W	EX-B2 (C2 and C6)	IW, 5HZ, 30 min
Jin 2020	RML	43 (29)	41 (24)	61.9 ± 5.7	62.7 ± 5.4	EA + CA + RDT	CA + RDT	siw, 3 W	2/5 under MS6 line, 2/5 under MS7 line	CW, 5HZ, 30 min
Wang 2014	RML	29 (14)	29 (16)	55.2 ± 5.6	56.5 ± 2.7	EA + IST + RDT	IST + RDT	siw, 4 W	Jia-lianquan	IW, 30 min
Peng 2015	RML	34 (NR)	34 (NR)	NR	NR	EA + RDT	RDT	qd, 20D	TE17	DW, 30 min
Yang 2022	SPSS, CE	30 (16)	30 (17)	70 ± 5	72 ± 5	EA + IST + ST	CA + IST + ST	siw, 3 W	CO15, TG3	IW, 1 mA, 5HZ, 30 min
Shao (a) 2022	RD	20 (12)	20 (16)	69.0 ± 12.96	63.5 ± 13.70	EA + ST + RDT	ST + RDT	qd, 4 W	TE17, GB20	CW, 2HZ, 30 min
Shao (b) 2022	RD	20 (15)	20 (17)	70.5 ± 13.70	63.0 ± 11.85	EA + CMT + ST + RDT	CMT + ST + RDT	qd, 4 W	TE17, GB20	CW, 2HZ, 30 min
Xin 2022	RML	60 (41)	30 (18)	62.82 ± 6.06	60.83 ± 5.93	EA/(EA + FN) + RDT	FN + RDT	siw, 4 W	GB20, Gongxue, GB12, EX-HN14	DW (2HZ/10HZ), 30 min
Peng 2022	RML	30 (16)	30 (17)	58.27 ± 4.127	59.23 ± 5.507	EA + CA + NES + ST + RDT	CA + NES + ST + RDT	siw, 4 W	TE17, GB20, ST4, ST6	CW, 30 min
Wang 2019	RML	46 (25)	45 (21)	60.89 ± 9.59	64.00 ± 9.81	EA + ST + IST + RDT	ST + IST + RDT	qd, 2 W	TE17, GB20	DW, 30 min

IG, interventional group; CG, controlled group; NR, not reported; RML, random number list; CE, closed envelope; SPSS: SPSS randomly divided; RD, randomly divided; EA, electroacupuncture; ST, swallowing training; CA, conventional acupuncture; RDT, routine drug treatment; NES, neuromuscular electrical stimulation; IST, ice stimulation training; CMT, Chinese medicine treatment; FN, float needle; siw, six times a week; bid, twice a day; qd, once a day; fiw, five times a week; DW, dilatational wave; IW, intermittent wave; CW, continuous wave.

### 3.3 Quality evaluation of the included literature

The Jadad scores of the articles included in this study are shown in Table 2. All 12 included articles mentioned randomized grouping, and 11 of them mentioned random number tables or similar methods. Five articles described randomization concealment through computer control or in other specific ways such as the use of sealed envelopes. Six articles stated that the trials were blinded, with three of them describing specific measures. Nine articles did not have subject dropout or described the number and reasons for subject dropout after it occurred.

**Table 2:** Jadad scores of the included studies.

Study	Randomization	Concealment of allocation	Blind methods	Withdrawals and dropouts	Total score
Chen, LF 2016	2	2	2	1	7
Zhang, ZL 2011	2	2	2	1	7
He, H 2018	2	2	2	1	7
Wang, JL 2014	2	2	1	0	5
Jin, HP 2020	2	1	0	1	4
Wang, LY 2014	2	1	0	1	4
Peng, YJ 2015	2	1	0	1	4
Yang, Y 2022	2	2	0	0	4
Shao, XZ 2022	1	1	1	1	4
Xin, GL 2022	2	1	0	1	4
Peng, YX 2022	2	1	1	0	4
Wang, Q 2019	2	1	0	1	4

**Figure 2:**
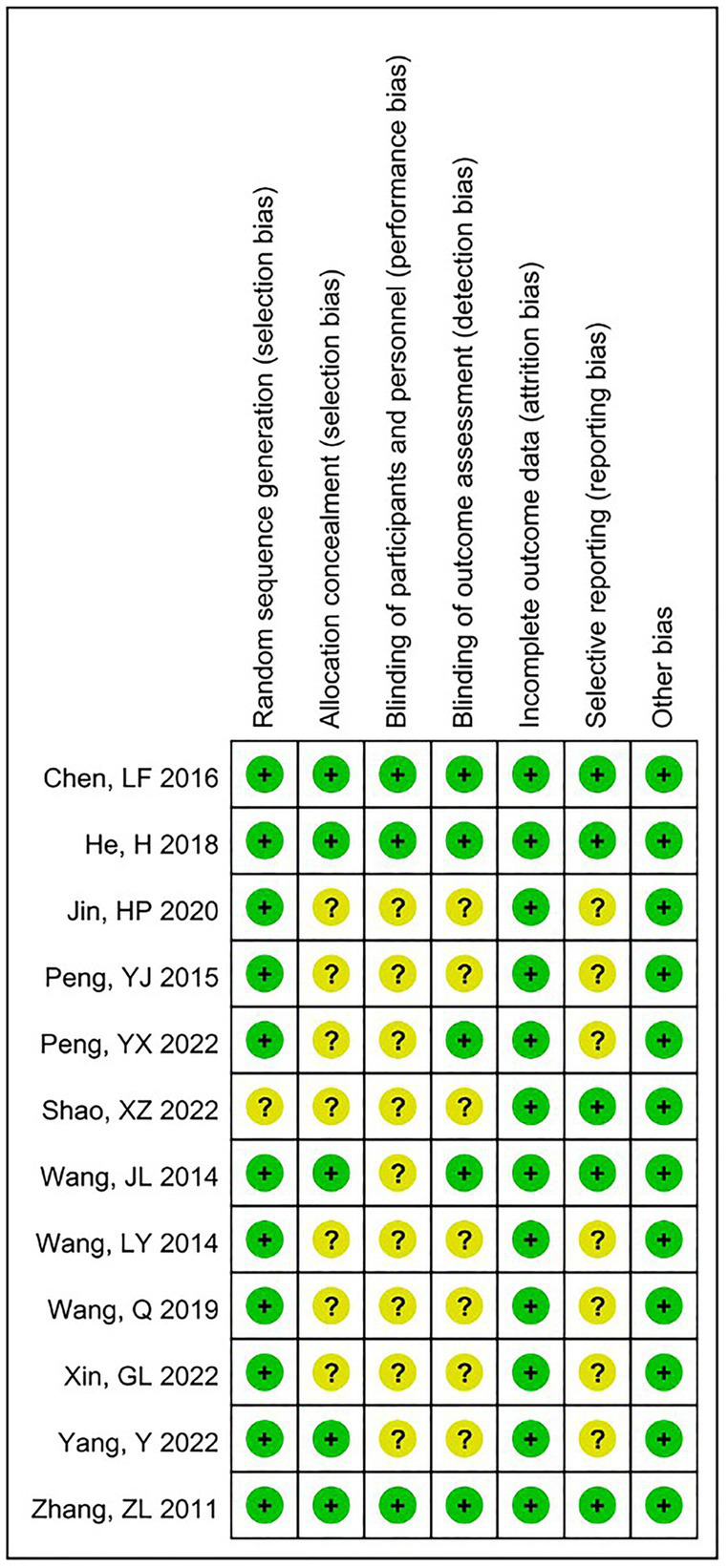
Methodological quality summary: review authors’ judgments about each study’s methodological quality.

**Figure 3:**
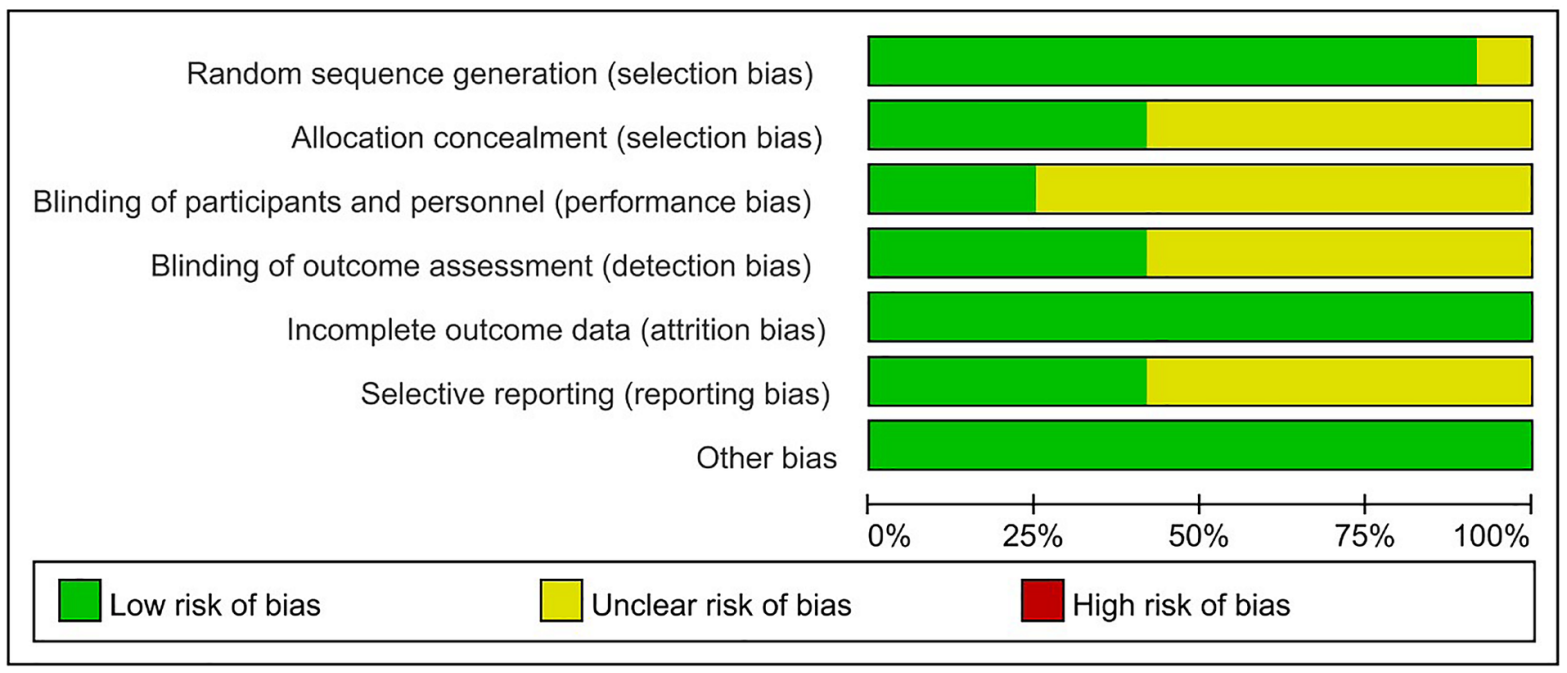
Methodological quality graph: review authors’ judgments about each study’s methodological quality.

**Figure 4:**
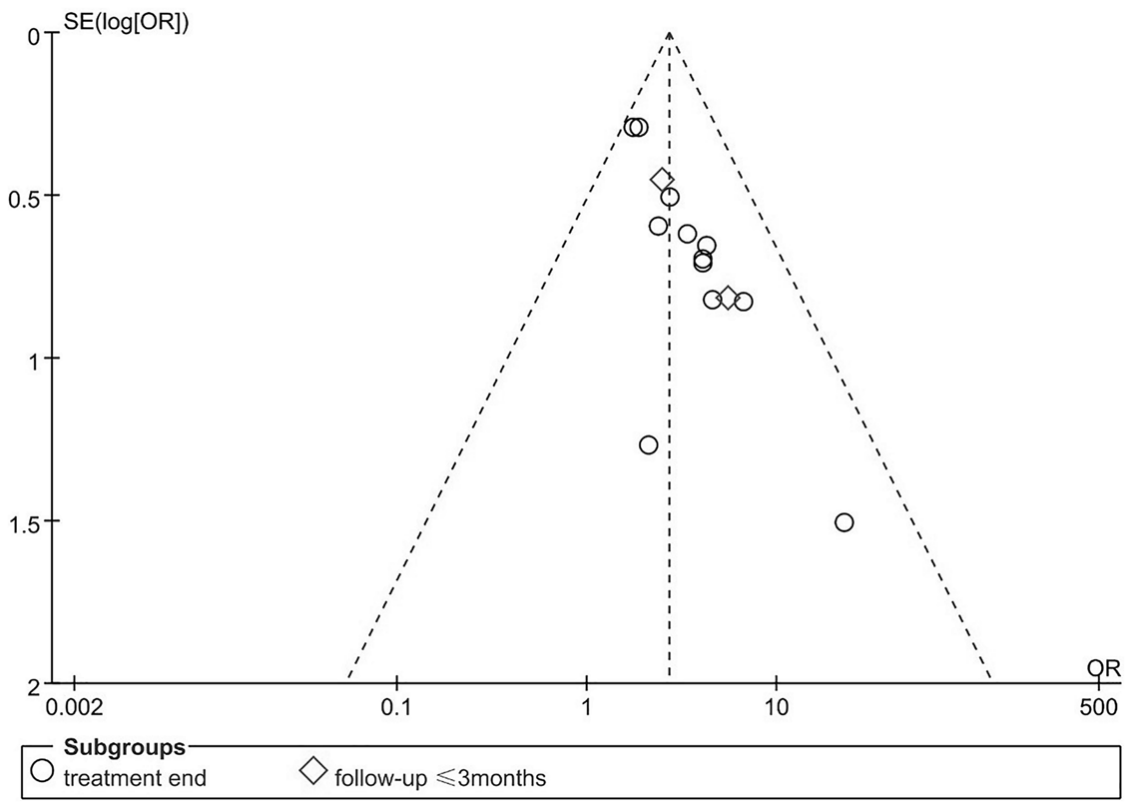
Publication bias of the involved studies.

The quality evaluation of the 12 included articles is shown in Figure 2 and Figure 3. Based on the Cochrane Collaboration’s tool for assessing the risk of bias, it was found that the risk of bias (14, 21, 23) was relatively low in three studies and unclear in nine studies (22, 24–31). As shown in Figure 4, a funnel plot was used to reflect the publication bias. The symmetrical curve graph showed that the publication bias of these studies was relatively low.

Finally, the evidence level of all outcome indicators was evaluated using the Grade Pro tool. The results showed that the evidence level of all outcome indicators remained at moderate or higher, with six outcome indicators assessed as high evidence level. The evidence level of each outcome indicator is shown in Supplementary File S2.

### 3.4 Primary outcome measures

#### 3.4.1 Clinical response rate

**Figure 5:**
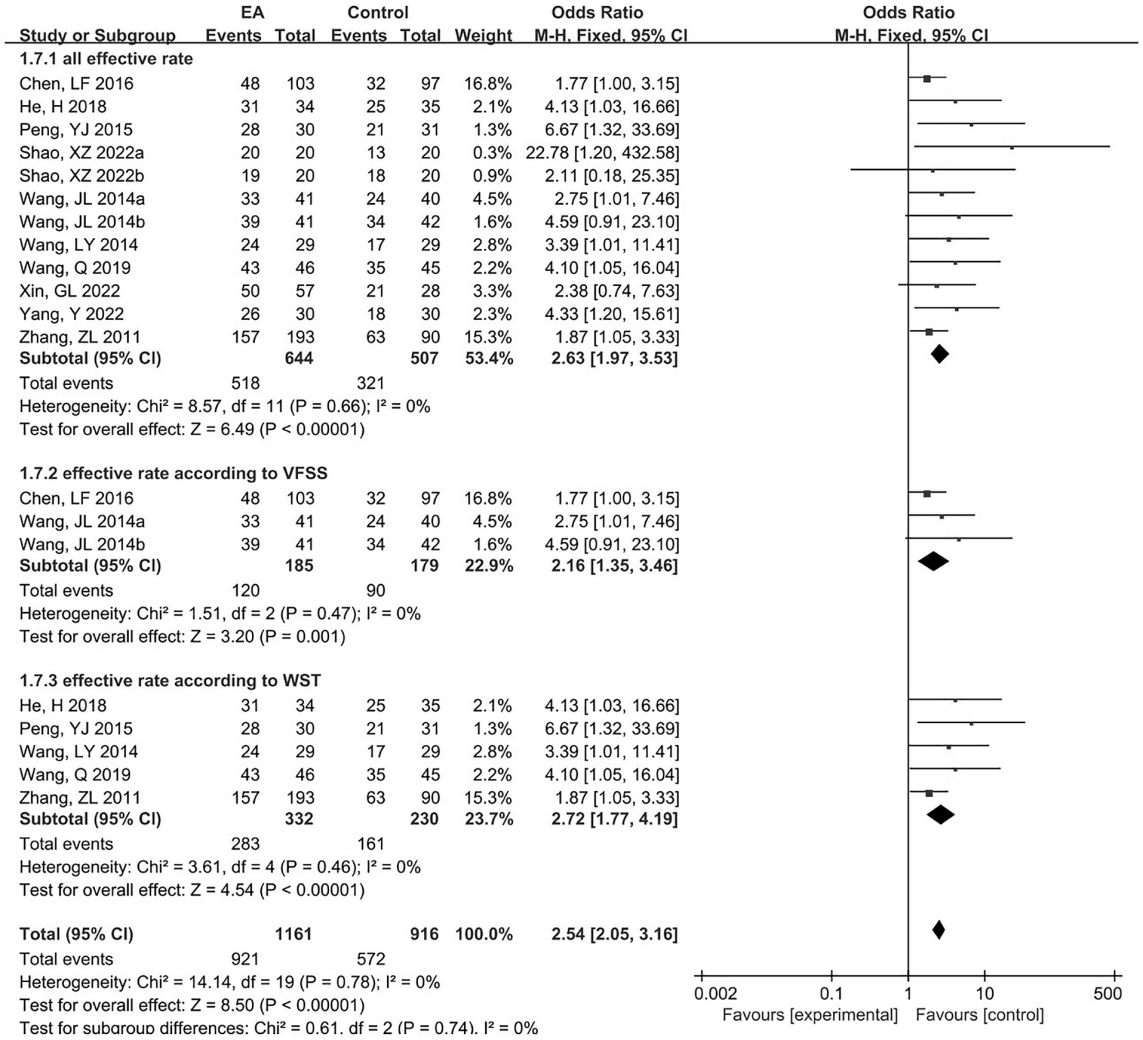
The forest plot of the effectiveness rate.

The clinical response rate was reported in 10 articles with 12 pairs of trials, including 644 subjects in the experimental group and 507 in the control group. A meta-analysis of 12 RCTs was performed using a fixed-effects model, and the results are shown in Figure 5. The results showed that the EA group was significantly more effective than the control group in terms of response rate (OR = 2.63, 95% CI = 1.97 to 3.53, *p* < 0.00001). Among them, the clinical response rate was reflected in three studies according to changes in the VFSS score. A subgroup analysis was conducted, indicating that the efficacy of the EA group was significantly superior to that of the control group (OR = 2.16, 95% CI = 1.35 to 3.46, *p* = 0.001). In five studies, the clinical response rate was reflected according to changes in the water swallow test (WST) score, revealing that the EA group had significantly higher efficacy than the control group (OR = 2.72, 95% CI = 1.77 to 4.19, *p* < 0.00001).

#### 3.4.2 VFSS scores

**Figure 6:**
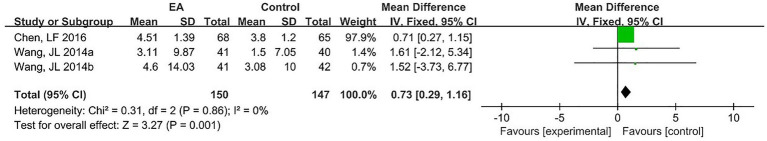
The forest plot for VFSS.

Two articles reported VFSS scores, including three pairs of trials, with 150 subjects in the experimental group and 147 in the control group. A meta-analysis of the three RCTs was performed using a fixed-effects model, and the results are shown in Figure 6. The results showed that the EA group showed more significant improvement in VFSS scores than the control group (MD = 0.73, 95% CI = 0.29 to 1.16, *p* = 0.001).

#### 3.4.3 SSA scores

**Figure 7:**
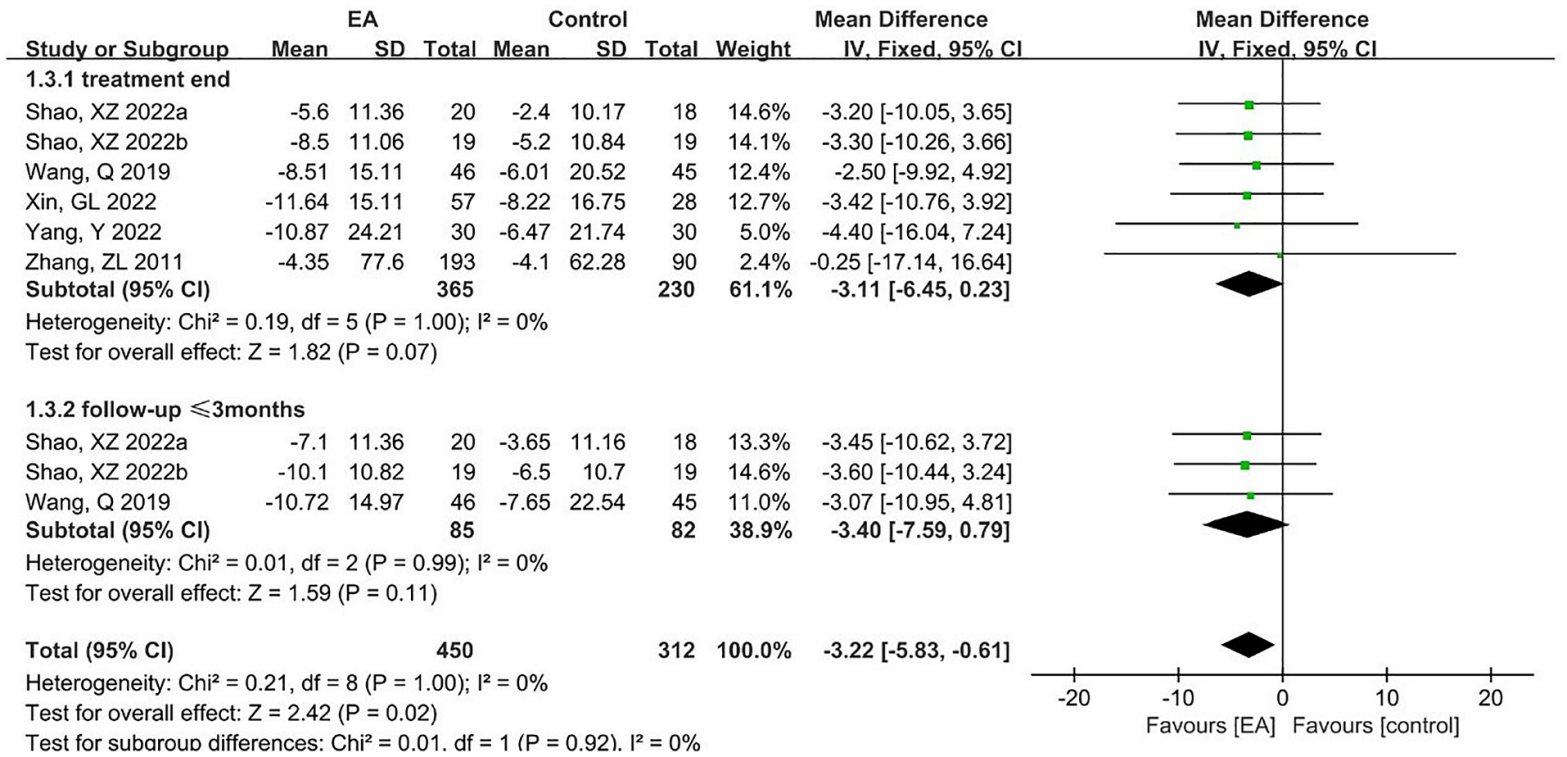
The forest plot for SSA.

Five articles reported SSA scores, including six pairs of trials, with 365 subjects in the experimental group and 230 in the control group. A meta-analysis of the six RCTs was conducted using a fixed-effects model, and the results are shown in Figure 7. The results showed that there was no statistical difference in SSA scores, although there was an improvement in the EA group (MD = -3.11, 95% CI = -6.45 to 0.23, *p* = 0.07). In addition, two articles (totaling three sets of trials) reported follow-up results. Subgroup analysis was performed and showed no statistical difference in SSA scores (MD = -3.40, 95% CI = -7.59 to 0.79, *p* = 0.11), but the combined effect size was statistically different (MD = -3.22, 95% CI = -5.83 to −0.61, *p* = 0.02).

#### 3.4.4 PAS scores

**Figure 8:**
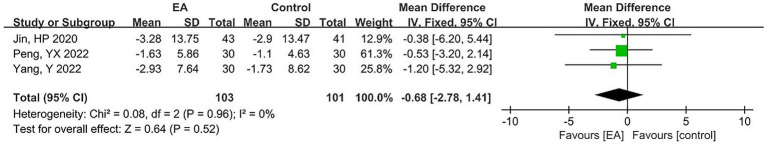
The forest plot for PAS.

Three articles reported PAS scores, with 103 subjects in the experimental group and 101 in the control group. A meta-analysis of the three RCTs was performed using a fixed-effects model, and the results are shown in Figure 8. The results showed that in terms of PAS scores, there was an improvement in the EA group but no statistical difference (MD = -0.68, 95% CI = -2.78 to 1.41, *p* = 0.52).

#### 3.4.5 SWAL-QOL scores

**Figure 9:**
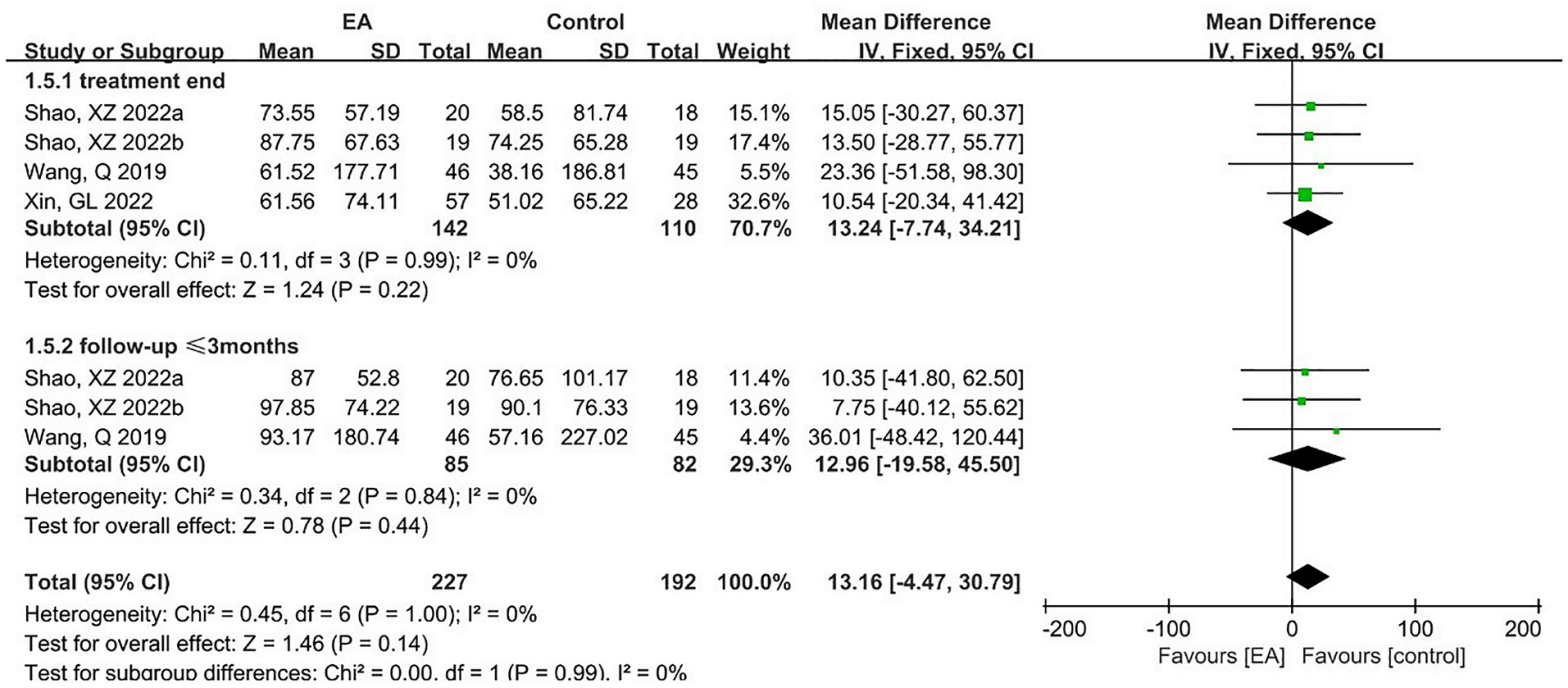
The forest plot for SWAL-QOL.

Three articles reported SWAL-QOL scores, including four pairs of trials, with 142 subjects in the experimental group and 110 in the control group. A meta-analysis of four RCTs was performed using a fixed-effects model, and the results are shown in Figure 9. The results showed that in terms of SWAL-QOL scores, there was an improvement in the EA group but no statistical difference (MD = 13.24, 95% CI = -7.74 to 34.21, *p* = 0.22). In addition, two articles (totaling three pairs of trials) reported follow-up results. Subgroup analysis was performed and showed no statistical difference in SWAL-QOL scores (MD = 12.96, 95% CI = -19.58 to 45.50, *p* = 0.44).

### 3.5 Secondary outcome indicators

#### 3.5.1 Adverse reactions

**Figure 10:**
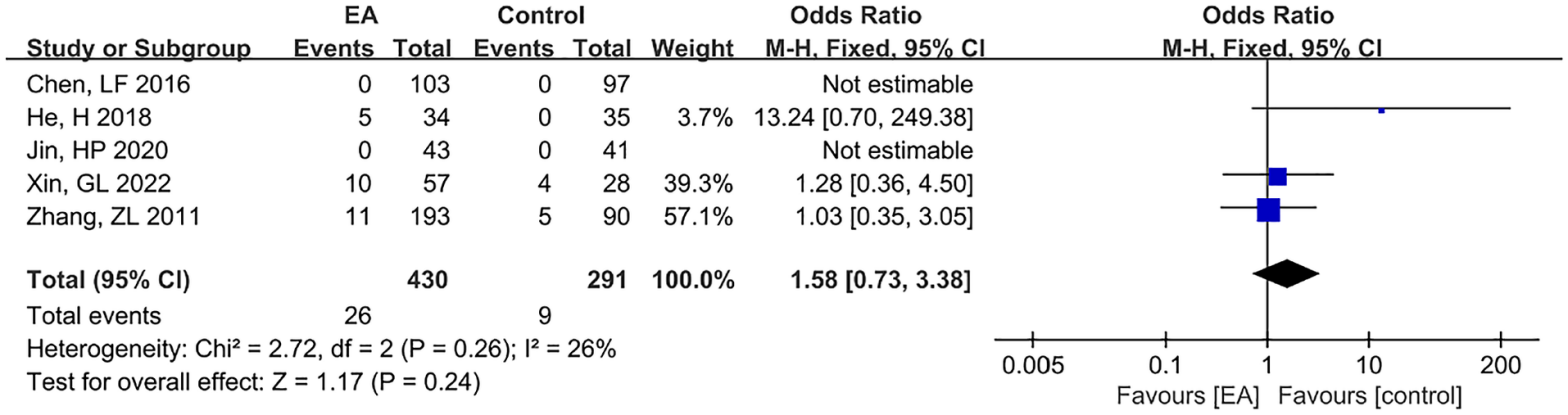
The forest plot for adverse events.

Adverse reactions were reported in seven articles, two of which were excluded because the specific number of subjects with adverse reactions was not described. The remaining five RCTs were subjected to meta-analysis, with 430 subjects in the experimental group and 291 in the control group. Adverse reactions included local numbness, subcutaneous hemorrhage, pain, and papular dermatitis. However, none of them were serious, and there were no life-threatening serious adverse events. A fixed-effects model was adopted for the meta-analysis, and the results of the meta-analysis are shown in Figure 10. The results showed no significant difference between the two groups in terms of adverse reactions, and EA did not increase the incidence of adverse reactions (OR = 1.58, 95% CI = 0.73 to 3.38, *p* = 0.24).

## 4 Discussion

This meta-analysis included 12 RCTs involving 1,358 patients with PSD. The results showed that EA could benefit patients with PSD in terms of the clinical response rate and VFSS score after the exclusion of low-quality literature and the inclusion of the latest studies.

Two meta-analyses (17, 32) previously published on the similar topic were also searched. In the study conducted by Huang JK et al. (17), although beneficial results were concluded, the authors pointed out that the quality of the included articles was relatively low. Accordingly, such results were not conclusive enough. Another meta-analysis published in a Chinese journal was a similar case (32), in which all the 18 articles included had a high risk of bias. Furthermore, some results of the two meta-analyses were significantly heterogeneous, but the reasons for heterogeneity were not discussed in either of them. These meta-analyses only included the articles on the comparison between EA combined with swallowing training and swallowing training alone, and excluded the studies on EA combined with other treatments. There is no study assessing whether the addition of EA will still be conducive to the rehabilitation of dysphagia when multiple interventions are combined. In our study, therefore, the studies on EA alone and EA combined with other interventions were included, while low-quality RCTs were excluded using the modified Jadad scale, which made our results more reliable and convincing. The assessment of evidence level also confirmed our judgment. The evidence level was high for six outcome indicators in this study, proving that the overall quality of this paper is high.

Based on our study results, there was no significant difference between both groups in the SSA score, PAS score, SWAL-QOL score, and incidence of adverse events. In terms of the clinical response rate and VFSS score, the improvement of the EA group was significantly superior to that of the control group. Our findings in the clinical response rate were the same as those concluded from previous meta-analyses (17, 32). However, the clinical response rate was not assessed using a unified measurement tool in previous meta-analyses. In our study, a subgroup analysis was conducted according to different measurement tools. Among the included studies, the clinical response rate was weighed according to changes in the VFSS score in three studies, while it was weighed based on the WST score in five studies. The results of the subgroup analysis still revealed that EA benefited patients and had relatively low heterogeneity. Therefore, our study was more credible from the perspective of the clinical response rate. Moreover, VFSS is the “gold standard” for the evaluation of dysphagia (33, 34). Based on our study findings, both the clinical response rate obtained from VFSS and the VFSS score showed that EA improved PSD. The efficacy of EA based on VFSS has also been demonstrated in the study conducted by Zhang Shengyu et al. (35), which is consistent with our observations.

Notably, EA did not improve the SSA score, PAS score, or SWAL-QOL score, which was inconsistent with the results of the clinical response rate and VFSS score. This may be related to different acupoint selections. It was found that in studies where the VFSS score was involved, the acupoint of Lianquan (CV23) was selected in all trials. In studies where the SSA score was involved, CV23 was selected in only one trial (21). In studies where PAS and SWAL-QOL scores were involved, it was not selected in any trial. The study conducted by Yuan S et al. (36) showed that administering EA on CV23 could regulate the swallowing function through excitatory neurons in the paraventricular hypothalamus. By comparing the effects of administering EA on CV23 and Neiguan (PC6), Ye QP et al. found that administering EA on CV23 could regulate the swallowing function by activating swallowing-related interneurons in the ventrolateral medulla oblongata (37). In addition, in a recent study, Ye QP et al. also demonstrated through optogenetics and chemogenetics that the NTS is involved in the regulation of EA on CV23 for PSD, revealing the role of the M1-NTS pathway in EA on CV23 (38). The latest research shows that in addition to the central nervous system, EA on CV23 can also have an impact on the peripheral nervous system and the local swallowing muscles. The study conducted by Yuan S et al. (39) suggests that TRPV1 at the CV23 can regulate local blood perfusion, thereby promoting the recovery of swallowing function. In combination with our study results, we believe that this may suggest a certain value of administering EA on CV23 in the improvement of PSD.

Additionally, we also recognize that the studies on EA in modern medicine have been increasingly getting advanced with the progress of science and technology. A growing number of articles are emerging to study and demonstrate the efficacy of EA with the aid of advanced equipment and technology. Wu Wenbao et al. (40) combined cerebral diffusion tensor imaging (DTI) techniques and VFSS to explore the clinical significance of acupuncture intervention in PSD. They administered EA on the acupoints of Sishencong, Baihui, Temple, Fengchi, and Tongue Triple Acupuncture and found that the fractional anisotropy (FA) values in the infarcted areas of the cerebral hemispheres were improved significantly, and that FA values were positively correlated with the integrity of white matter fiber tracts (41), suggesting that EA on relevant acupoints may have restored cortical function. Zhu Runjia et al. (42) observed the clinical efficacy of EA in the treatment of ischemic stroke through transcranial Doppler (TCD), finding that EA stimulation could improve the intracranial vascular function of patients. The main action principle is to reduce vascular resistance by changing the elasticity and compliance of the corresponding blood vessel, so as to increase cerebral blood supply and promote rehabilitation after stroke, thus indirectly improving the swallowing function. Lan Chunwei et al. (43) observed the effect of EA on PSD through magnetic resonance spectroscopy (MRS) and surface electromyography (sEMG), finding that EA could decrease the contents of rNAA and Lac/Cr in the brain to reduce the mean amplitude of sEMG in the pharynx and shorten the swallowing time course. Their findings indicate that EA improves the swallowing function of patients with PSD by influencing brain metabolism and ameliorating the contraction of peripheral swallowing muscles. Most of the outcome indicators included in our study were scale scores. In fact, we also wanted to analyze the efficacy of EA in combination with microcosmic study data. However, it was a pity that among our included studies, EEG was performed in only one study to detect the degree of cerebral cortex inhibition (24); a transcranial magnetic stimulator was used in one study to detect the effect of EA on motor-evoked potential (MEP) (27), and sEMG was performed in one study to quantitatively assess swallowing muscle groups (29). In this context, we cannot provide further evidence to explore the efficacy of EA. More high-quality studies are desired to solve this problem in the future.

We also acknowledge that this systematic review has some limitations. Firstly, based on the Cochrane Collaboration’s tool for assessing the risk of bias, nine of our included studies (22, 24–31) had unclear risk of bias. For example, Shao XZ et al. (28) only expressed random allocation but did not describe the specific method for random allocation, which reduced the quality of this meta-analysis. Secondly, some outcome indicators were only involved in two to three studies. In this regard, the insufficient sample size was also one of our regrets. Finally, it was impossible to continue the efficacy analysis of EA from the perspective of new technology since there were few studies reporting microcosmic study data.

In spite of some limitations, the findings of this systematic review are still of great clinical and basic scientific significance. In combination with previous meta-analyses (17, 32), we are surer that EA is a safe and reliable treatment approach for patients with PSD. Although further studies are still required to demonstrate the efficacy of EA (acupoint selection in EA or with the aid of new technology), this paper still provides a new evidence-based basis for the clinical rehabilitation of patients with PSD and offers the orientation and theoretical support for future studies. Therefore, our study is indeed of great value.

## 5 Conclusion

In conclusion, EA is effective and safe in treating PSD. EA combined with conventional treatment or other interventions can significantly improve the clinical response rate and VFSS score in patients with PSD, without increasing the incidence of adverse reactions. There is no statistical difference between the two groups in terms of the improvement of SSA score, PAS score, and SWAL-QOL score. More high-quality RCT studies are still needed in the future to further explore the efficacy of EA in the treatment of PSD.

## Data availability statement

The original contributions presented in the study are included in the article/Supplementary material, further inquiries can be directed to the corresponding author.

## Author contributions

XL: Formal analysis, Investigation, Writing – original draft, Writing – review & editing. HL: Formal analysis, Investigation, Writing – original draft. WY: Methodology, Writing – original draft. HG: Formal analysis, Investigation, Writing – review & editing. KG: Methodology, Writing – review & editing. LL: Writing – review & editing. XF: Writing – review & editing. ZH: Conceptualization, Writing – review & editing.

## Glossary

EA: electroacupuncture
PSD: poststroke dysphagia
RCTs: randomized controlled trials
VFSS: videofluoroscopic swallowing study
SSA: standardized swallowing assessment
PAS: Rosenbek penetration-aspiration scale
SWAL-QOL: Swallowing Quality of Life
USD: USA dollar
NTS: nucleus tractus solitarius
NA: nucleus ambiguus
M1: primary motor cortex
PBN: parabrachial nucleus
FEES: fiberoptic endoscopic evaluation of swallowing
OR: odds ratio
CI: confidence interval
MD: mean difference
WST: water swallow test
CV23: Lianquan
PC6: Neiguan
DTI: diffusion tensor imaging
FA: fractional anisotropy
TCD: transcranial Doppler
MRS: magnetic resonance spectroscopy
sEMG: surface electromyography
rNAA: The concentrations of N-acetylaspartate (NAA) in the infarct lesion/NAA in the contralateral mirror site ratio
Lac: lactic acid
Cr: creatine
EEG: electroencephalography
MEP: motor-evoked potential
