# Corrigendum: Systematic review and meta-analysis of the efficacy and safety of electroacupuncture for poststroke dysphagia

**DOI:** 10.3389/fneur.2023.1359704

**Published:** 2024-01-10

**Authors:** Xuezheng Li, Lijun Lu, Xuefeng Fu, Hao Li, Wen Yang, Hua Guo, Kaifeng Guo, Zhen Huang

**Affiliations:** ^1^Postgraduate Cultivation Base of Guangzhou University of Chinese Medicine, Panyu Central Hospital, Guangzhou, Guangdong, China; ^2^Department of Rehabilitation Medicine, Guangzhou Panyu Central Hospital, Guangzhou Guangdong, China

**Keywords:** stroke, dysphagia, electroacupuncture, meta-analysis, systematic review, randomized controlled trials

In the published article, there was an error in affiliation 1. Instead of “Guangzhou University of Chinese Medicine, Guangzhou, Guangdong, China”, it should be “Postgraduate Cultivation Base of Guangzhou University of Chinese Medicine, Panyu Central Hospital, Guangzhou, Guangdong, China”.

The authors apologize for this error and state that this does not change the scientific conclusions of the article in any way. The original article has been updated.

