# Supplementary material for: Systematic review and meta-analysis of the efficacy and safety of electroacupuncture for poststroke dysphagia
Source: Front Neurol. 2023 Dec 6;14:1270624. doi: 10.3389/fneur.2023.1270624 (PMC10731355; doi:10.3389/fneur.2023.1270624)
Supplement: Supplementary file 1 [file Data_Sheet_1.docx]

***Supplementary Material***

**Supplementary File 1 search strategy**

**Table 1:** PubMed Search

| **NO** | **Search Details** | **Results** |
| --- | --- | --- |
| #1 | "Electroacupuncture"[MeSH Terms] | 4,798 |
| #2 | "Stroke"[MeSH Terms] | 167,536 |
| #3 | "Deglutition Disorders"[MeSH Terms] | 58,574 |
| #4 | (("acupunctural"[All Fields] OR "acupuncture"[MeSH Terms] OR "acupuncture"[All Fields] OR "acupuncture therapy"[MeSH Terms] OR ("acupuncture"[All Fields] AND "therapy"[All Fields]) OR "acupuncture therapy"[All Fields] OR "acupuncture s"[All Fields] OR "acupunctured"[All Fields] OR "acupunctures"[All Fields] OR "acupuncturing"[All Fields]) AND ("electricity"[MeSH Terms] OR "electricity"[All Fields] OR "electric"[All Fields] OR "electrical"[All Fields] OR "electrically"[All Fields] OR "electrics"[All Fields])) OR (("electricity"[MeSH Terms] OR "electricity"[All Fields] OR "electric"[All Fields] OR "electrical"[All Fields] OR "electrically"[All Fields] OR "electrics"[All Fields]) AND ("acupunctural"[All Fields] OR "acupuncture"[MeSH Terms] OR "acupuncture"[All Fields] OR "acupuncture therapy"[MeSH Terms] OR ("acupuncture"[All Fields] AND "therapy"[All Fields]) OR "acupuncture therapy"[All Fields] OR "acupuncture s"[All Fields] OR "acupunctured"[All Fields] OR "acupunctures"[All Fields] OR "acupuncturing"[All Fields])) OR (("electricity"[MeSH Terms] OR "electricity"[All Fields] OR "electric"[All Fields] OR "electrical"[All Fields] OR "electrically"[All Fields] OR "electrics"[All Fields]) AND ("acupoint s"[All Fields] OR "acupuncture points"[MeSH Terms] OR ("acupuncture"[All Fields] AND "points"[All Fields]) OR "acupuncture points"[All Fields] OR "acupoint"[All Fields] OR "acupoints"[All Fields]) AND ("stimulate"[All Fields] OR "stimulated"[All Fields] OR "stimulates"[All Fields] OR "stimulating"[All Fields] OR "stimulation"[All Fields] OR "stimulations"[All Fields] OR "stimulative"[All Fields] OR "stimulator"[All Fields] OR "stimulator s"[All Fields] OR "stimulators"[All Fields])) OR (("electricity"[MeSH Terms] OR "electricity"[All Fields] OR "electric"[All Fields] OR "electrical"[All Fields] OR "electrically"[All Fields] OR "electrics"[All Fields]) AND ("acupunctural"[All Fields] OR "acupuncture"[MeSH Terms] OR "acupuncture"[All Fields] OR "acupuncture therapy"[MeSH Terms] OR ("acupuncture"[All Fields] AND "therapy"[All Fields]) OR "acupuncture therapy"[All Fields] OR "acupuncture s"[All Fields] OR "acupunctured"[All Fields] OR "acupunctures"[All Fields] OR "acupuncturing"[All Fields])) OR "electro-acupuncture"[All Fields] OR (("electrode s"[All Fields] OR "electroded"[All Fields] OR "electrodes"[MeSH Terms] OR "electrodes"[All Fields] OR "electrode"[All Fields] OR "electrodic"[All Fields]) AND ("acupunctural"[All Fields] OR "acupuncture"[MeSH Terms] OR "acupuncture"[All Fields] OR "acupuncture therapy"[MeSH Terms] OR ("acupuncture"[All Fields] AND "therapy"[All Fields]) OR "acupuncture therapy"[All Fields] OR "acupuncture s"[All Fields] OR "acupunctured"[All Fields] OR "acupunctures"[All Fields] OR "acupuncturing"[All Fields])) OR (("electronical"[All Fields] OR "electronically"[All Fields] OR "electronics"[MeSH Terms] OR "electronics"[All Fields] OR "electronic"[All Fields]) AND ("acupunctural"[All Fields] OR "acupuncture"[MeSH Terms] OR "acupuncture"[All Fields] OR "acupuncture therapy"[MeSH Terms] OR ("acupuncture"[All Fields] AND "therapy"[All Fields]) OR "acupuncture therapy"[All Fields] OR "acupuncture s"[All Fields] OR "acupunctured"[All Fields] OR "acupunctures"[All Fields] OR "acupuncturing"[All Fields])) | 7,719 |
| #5 | "stroke"[MeSH Terms] OR "stroke"[All Fields] OR "strokes"[All Fields] OR "stroke s"[All Fields] OR ("stroke"[MeSH Terms] OR "stroke"[All Fields] OR ("cerebrovascular"[All Fields] AND "accident"[All Fields]) OR "cerebrovascular accident"[All Fields]) OR ("stroke"[MeSH Terms] OR "stroke"[All Fields] OR ("cerebrovascular"[All Fields] AND "accidents"[All Fields]) OR "cerebrovascular accidents"[All Fields]) OR (("stroke"[MeSH Terms] OR "stroke"[All Fields] OR "cva"[All Fields]) AND ("stroke"[MeSH Terms] OR "stroke"[All Fields] OR ("cerebrovascular"[All Fields] AND "accident"[All Fields]) OR "cerebrovascular accident"[All Fields])) OR ("CVAs"[All Fields] AND ("stroke"[MeSH Terms] OR "stroke"[All Fields] OR ("cerebrovascular"[All Fields] AND "accident"[All Fields]) OR "cerebrovascular accident"[All Fields])) OR ("stroke"[MeSH Terms] OR "stroke"[All Fields] OR ("cerebrovascular"[All Fields] AND "apoplexy"[All Fields]) OR "cerebrovascular apoplexy"[All Fields]) OR ("stroke"[MeSH Terms] OR "stroke"[All Fields] OR ("brain"[All Fields] AND "vascular"[All Fields] AND "accident"[All Fields]) OR "brain vascular accident"[All Fields]) OR ("stroke"[MeSH Terms] OR "stroke"[All Fields] OR ("brain"[All Fields] AND "vascular"[All Fields] AND "accidents"[All Fields]) OR "brain vascular accidents"[All Fields]) OR ("stroke"[MeSH Terms] OR "stroke"[All Fields] OR ("cerebrovascular"[All Fields] AND "stroke"[All Fields]) OR "cerebrovascular stroke"[All Fields]) OR ("stroke"[MeSH Terms] OR "stroke"[All Fields] OR ("cerebrovascular"[All Fields] AND "strokes"[All Fields]) OR "cerebrovascular strokes"[All Fields]) OR ("apoplexies"[All Fields] OR "stroke"[MeSH Terms] OR "stroke"[All Fields] OR "apoplexy"[All Fields]) OR ("stroke"[MeSH Terms] OR "stroke"[All Fields] OR ("cerebral"[All Fields] AND "stroke"[All Fields]) OR "cerebral stroke"[All Fields]) OR ("stroke"[MeSH Terms] OR "stroke"[All Fields] OR ("cerebral"[All Fields] AND "strokes"[All Fields]) OR "cerebral strokes"[All Fields]) OR ("stroke"[MeSH Terms] OR "stroke"[All Fields] OR ("stroke"[All Fields] AND "cerebral"[All Fields]) OR "stroke cerebral"[All Fields]) OR ("stroke"[MeSH Terms] OR "stroke"[All Fields] OR ("strokes"[All Fields] AND "cerebral"[All Fields]) OR "strokes cerebral"[All Fields]) OR ("stroke"[MeSH Terms] OR "stroke"[All Fields] OR ("acute"[All Fields] AND "stroke"[All Fields]) OR "acute stroke"[All Fields]) OR ("stroke"[MeSH Terms] OR "stroke"[All Fields] OR ("acute"[All Fields] AND "strokes"[All Fields]) OR "acute strokes"[All Fields]) OR ("stroke"[MeSH Terms] OR "stroke"[All Fields] OR ("acute"[All Fields] AND "cerebrovascular"[All Fields] AND "accident"[All Fields]) OR "acute cerebrovascular accident"[All Fields]) OR ("stroke"[MeSH Terms] OR "stroke"[All Fields] OR ("acute"[All Fields] AND "cerebrovascular"[All Fields] AND "accidents"[All Fields]) OR "acute cerebrovascular accidents"[All Fields]) | 431,706 |
| #6 | "deglutition disorders"[MeSH Terms] OR ("deglutition"[All Fields] AND "disorders"[All Fields]) OR "deglutition disorders"[All Fields] OR ("deglutition"[All Fields] AND "disorder"[All Fields]) OR "deglutition disorder"[All Fields] OR ("deglutition disorders"[MeSH Terms] OR ("deglutition"[All Fields] AND "disorders"[All Fields]) OR "deglutition disorders"[All Fields] OR ("disorders"[All Fields] AND "deglutition"[All Fields]) OR "disorders deglutition"[All Fields]) OR ("deglutition disorders"[MeSH Terms] OR ("deglutition"[All Fields] AND "disorders"[All Fields]) OR "deglutition disorders"[All Fields] OR ("swallowing"[All Fields] AND "disorders"[All Fields]) OR "swallowing disorders"[All Fields]) OR ("deglutition disorders"[MeSH Terms] OR ("deglutition"[All Fields] AND "disorders"[All Fields]) OR "deglutition disorders"[All Fields] OR ("swallowing"[All Fields] AND "disorder"[All Fields]) OR "swallowing disorder"[All Fields]) OR ("deglutition disorders"[MeSH Terms] OR ("deglutition"[All Fields] AND "disorders"[All Fields]) OR "deglutition disorders"[All Fields] OR "dysphagia"[All Fields] OR "dysphagias"[All Fields]) OR ("deglutition disorders"[MeSH Terms] OR ("deglutition"[All Fields] AND "disorders"[All Fields]) OR "deglutition disorders"[All Fields] OR ("oropharyngeal"[All Fields] AND "dysphagia"[All Fields]) OR "oropharyngeal dysphagia"[All Fields]) OR ("deglutition disorders"[MeSH Terms] OR ("deglutition"[All Fields] AND "disorders"[All Fields]) OR "deglutition disorders"[All Fields] OR ("esophageal"[All Fields] AND "dysphagia"[All Fields]) OR "esophageal dysphagia"[All Fields]) | 79,778 |
| #7 | #1 or #4 | 11,014 |
| #8 | #2 or #5 | 431,706 |
| #9 | #3 or #6 | 79,778 |
| #10 | #8 and #9 | 3,164 |
| #11 | #7 and #10 | 38 |

**Table 2:** EMbase Search

| **NO** | **Search Details** | **Results** |
| --- | --- | --- |
| #1 | 'electroacupuncture'/exp OR electroacupuncture OR (acupuncture, AND electric) OR (electric AND acupuncture) OR (electrical AND acupoint AND stimulation) OR (electrical AND acupuncture) OR 'electro acupuncture' OR (electrode AND acupuncture) OR (electronic AND acupuncture) | 34,518 |
| #2 | 'cerebrovascular accident'/exp OR 'cerebrovascular accident' OR (cerebrovascular AND ('accident'/exp OR accident)) OR (accident, AND cerebrovascular) OR (acute AND cerebrovascular AND lesion) OR (acute AND focal AND cerebral AND vasculopathy) OR (acute AND stroke) OR (apoplectic AND stroke) OR apoplexia OR apoplexy OR (blood AND flow AND disturbance, AND brain) OR (brain AND accident) OR (brain AND attack) OR (brain AND blood AND flow AND disturbance) OR (brain AND insult) OR (brain AND insultus) OR (brain AND vascular AND accident) OR (cerebral AND apoplexia) OR (cerebral AND insult) OR (cerebral AND stroke) OR (cerebral AND vascular AND accident) OR (cerebral AND vascular AND insufficiency) OR (cerebro AND vascular AND accident) OR (cerebrovascular AND arrest) OR (cerebrovascular AND failure) OR (cerebrovascular AND injury) OR (cerebrovascular AND insufficiency) OR (cerebrovascular AND insult) OR (cerebrum AND vascular AND accident) OR (cryptogenic AND stroke) OR cva OR (insultus AND cerebralis) OR (ischaemic AND seizure) OR (ischemic AND seizure) OR stroke OR (thrombotic AND stroke) | 731,854 |
| #3 | 'dysphagia'/exp OR dysphagia OR aphagopraxia OR (deglutition AND difficulty) OR (deglutition AND disorder) OR (deglutition AND disorders) OR (difficult AND deglutition) OR (difficulty AND in AND swallowing) OR (difficulty AND swallowing) OR dysphagias OR (swallowing AND difficult) OR (swallowing AND difficultness) OR (swallowing AND difficulty) OR (swallowing AND disorder) | 111,600 |
| #4 | #2 AND #3 | 7,978 |
| #5 | #1 AND #4 | 111 |

**Table 3:** Web of Science Search

| **NO** | **Search Details** | **Results** |
| --- | --- | --- |
| #1 | "((((((TS=(Electroacupuncture)) OR TS=(electric acupuncture)) OR TS=(electrical acupoint stimulation)) OR TS=(electrical acupuncture)) OR TS=(electro-acupuncture)) OR TS=(electrode acupuncture)) OR TS=(electronic acupuncture) " | 24,110 |
| #2 | "(((((((((((((((((((TS=(stroke)) OR TS=(Strokes)) OR TS=(Cerebrovascular Accident)) OR TS=(Cerebrovascular Accidents)) OR TS=(CVA)) OR TS=(CVAs)) OR TS=(Cerebrovascular Apoplexy)) OR TS=(Brain Vascular Accident)) OR TS=(Brain Vascular Accidents)) OR TS=(Cerebrovascular Stroke)) OR TS=(Cerebrovascular Strokes)) OR TS=(Apoplexy)) OR TS=(Cerebral Stroke)) OR TS=(Cerebral Strokes)) OR TS=(Stroke, Cerebral)) OR TS=(Strokes, Cerebral)) OR TS=(Acute Stroke)) OR TS=(Acute Strokes)) OR TS=(Acute Cerebrovascular Accident)) OR TS=(Acute Cerebrovascular Accidents) " | 872,965 |
| #3 | "(((((((TS=(Deglutition Disorders)) OR TS=(Deglutition Disorder)) OR TS=(Disorders, Deglutition)) OR TS=(Swallowing Disorders)) OR TS=(Swallowing Disorder)) OR TS=(Dysphagia)) OR TS=(Oropharyngeal Dysphagia)) OR TS=(Esophageal Dysphagia) " | 70,566 |
| #4 | #2 AND #3 | 6,136 |
| #5 | #1 AND #4 | 82 |

**Table 4:** Cochrane Library Search

| **NO** | **Search Details** | **Results** |
| --- | --- | --- |
| #1 | MeSH descriptor: [Electroacupuncture] explode all trees | 1,003 |
| #2 | MeSH descriptor: [Stroke] explode all trees | 14,103 |
| #3 | MeSH descriptor: [Deglutition Disorders] explode all trees | 3,512 |
| #4 | (Strokes):ti,ab,kw OR (Cerebrovascular Accident):ti,ab,kw OR (Cerebrovascular Accidents):ti,ab,kw OR (CVA):ti,ab,kw OR (CVAs):ti,ab,kw | 69,896 |
| #5 | (Cerebrovascular Apoplexy):ti,ab,kw OR (Brain Vascular Accident):ti,ab,kw OR (Brain Vasculan Accidents):ti,ab,kw OR (Cerebrovascular Stroke):ti,ab,kw OR (Cerebrovascular Strokes):ti,ab,kw | 16,225 |
| #6 | (Apoplexy):ti,ab,kw OR (Cerebral Stroke):ti,ab,kw OR (Cerebral Strokes):ti,ab,kw OR (Stroke, Cerebral):ti,ab,kw OR (Strokes, Cerebra):ti,ab,kw | 7,739 |
| #7 | (Acute Stroke):ti,ab,kw OR (Acute Strokest):ti,ab,kw OR (Acute Cerebrovascular Accident):ti,ab,kw OR (Acute Cerebrovascular Accients):ti,ab,kw | 18,432 |
| #8 | (acupuncture, electric):ti,ab,kw OR (electric acupuncture):ti,ab,kw OR (electrical acupoint stimulatin):ti,ab,kw OR (electrical acupuncture):ti,ab,kw OR (electroacupuncture):ti,ab,kw | 2,439 |
| #9 | (electrode acupuncture):ti,ab,kw OR (electronic acupuncture):ti,ab,kw | 382 |
| #10 | (Deglutition Disorder):ti,ab,kw OR (Disorders, Dgltition):ti,ab,kw OR (Swallowing Disorders):ti,ab,kw OR (Swallowing Disorder):ti,ab,kw OR (Dysphagia):ti,ab,kw | 5,890 |
| #11 | (Oropharyngeal Dysphagia):ti,ab,kw OR (Esophageal Dysphagia):ti,ab,kw | 1,614 |
| #12 | #1 or #8 or #9 | 3,296 |
| #13 | #2 or #4 or #5 or #6 or #7 | 70,624 |
| #14 | #3 or #10 or #11 | 8,062 |
| #15 | #12 and #13 and #14 | 32 |

**Table 5:** CNKI Search

| **NO** | **Search Details** | **Results** |
| --- | --- | --- |
| #1 | (篇关摘:电针(精确) ) OR (篇关摘:电针疗法(精确) ) AND ( (篇关摘:卒中(精确)) OR (篇关摘:中风(精确)) OR (篇关摘:脑血管意外(精确) ) OR (篇关摘:脑梗死(精确)) OR(篇关摘:脑出血(精确) ) OR (篇关摘:脑栓塞(精确)) OR(篇关摘:脑缺血(精确))OR(篇关摘:脑血管闭塞(精确) ) OR (篇关摘:脑血栓(精确) ) OR (篇关摘: 蛛网膜下腔出血(精确)))AND((篇关摘:吞咽障碍(精确))OR(篇关摘:吞咽困难(精确) ) OR (篇关摘:吞咽功能障碍(精确)) ) | 310 |

**Table 6:** Wanfang Search

| **NO** | **Search Details** | **Results** |
| --- | --- | --- |
| #1 | 主题:(电针) or 主题:(电针疗法) | 27,588 |
| #2 | 主题:(卒中) or 主题:(中风) or 主题:(脑血管意外) or 主题:(脑梗死) or 主题:(脑出血) or 主题:(脑栓塞) | 443,367 |
| #3 | 主题:(脑缺血) or 主题:(脑血管闭塞) or 主题:(脑血栓) or 主题:(蛛网膜下腔出血) | 87,553 |
| #4 | 主题:(吞咽障碍) or 主题:(吞咽困难) or 主题:(吞咽功能障碍) | 29,175 |
| #5 | #2 or #3 | 470,501 |
| #6 | #1 and #5 and #4 | 305 |

**Table 7:** CBM Search

| NO | Search Details | Results |
| --- | --- | --- |
| #1 | "电针"[常用字段:智能] OR "电针疗法"[常用字段:智能] | 33,938 |
| #2 | "卒中"[常用字段:智能] OR "中风"[常用字段:智能] OR "脑血管意外"[常用字段:智能] OR "脑梗死"[常用字段:智能] OR "脑出血"[常用字段:智能] OR "脑栓塞"[常用字段:智能] OR "脑缺血"[常用字段:智能] OR "脑血管闭塞"[常用字段:智能] OR "蛛网膜下腔出血"[常用字段:智能] | 545,986 |
| #3 | "吞咽障碍"[常用字段:智能] OR "吞咽困难"[常用字段:智能] OR "吞咽功能障碍"[常用字段:智能] | 103,331 |
| #4 | #1 and #2 and #3 | 238 |
