# Supplementary material for: Systematic review and meta-analysis of the efficacy and safety of electroacupuncture for poststroke dysphagia
Source: Front Neurol. 2023 Dec 6;14:1270624. doi: 10.3389/fneur.2023.1270624 (PMC10731355; doi:10.3389/fneur.2023.1270624)
Supplement: Supplementary file 2 [file Data_Sheet_2.docx]

***Supplementary Material***

**Supplementary File 2** grade pro assessment results

| **EA FOR PSD** | | | | | | |
| --- | --- | --- | --- | --- | --- | --- |
| **Patient or population:** patients with PSD **Settings:**  **Intervention:** EA FOR PSD | | | | | | |
| **Outcomes** | **Illustrative comparative risks* (95% CI)** | | **Relative effect (95% CI)** | **No of Participants (studies)** | **Quality of the evidence (GRADE)** | **Comments** |
|  | Assumed risk | Corresponding risk |  |  |  |  |
|  | **Control** | **EA FOR PSD** |  |  |  |  |
| **VFSS change** |  | The mean vfss change in the intervention groups was **0.73 higher** (0.29 to 1.16 higher) |  | 297 (3 studies) | ⊕⊕⊕⊝ **moderate**^1^ |  |
| **SSA change** |  | The mean ssa change in the intervention groups was **3.22 lower** (5.83 to 0.61 lower) |  | 762 (6 studies) | ⊕⊕⊕⊝ **moderate**^2^ |  |
| **SSA change - treatment end** |  | The mean ssa change - treatment end in the intervention groups was **3.11 lower** (6.45 lower to 0.23 higher) |  | 595 (6 studies) | ⊕⊕⊕⊕ **high** |  |
| **SSA change - follow-up ≤3months** |  | The mean ssa change - follow-up ≤3months in the intervention groups was **3.4 lower** (7.59 lower to 0.79 higher) |  | 167 (3 studies) | ⊕⊕⊕⊝ **moderate**^1^ |  |
| **Rosenbek change** |  | The mean rosenbek change in the intervention groups was **0.68 lower** (2.78 lower to 1.41 higher) |  | 204 (3 studies) | ⊕⊕⊕⊝ **moderate**^1^ |  |
| **SWAL-QOL change** |  | The mean swal-qol change in the intervention groups was **13.16 higher** (4.47 lower to 30.79 higher) |  | 419 (4 studies) | ⊕⊕⊕⊝ **moderate**^1^ |  |
| **SWAL-QOL change - treatment end** |  | The mean swal-qol change - treatment end in the intervention groups was **13.24 higher** (7.74 lower to 34.21 higher) |  | 252 (4 studies) | ⊕⊕⊕⊝ **moderate**^1^ |  |
| **SWAL-QOL change - follow-up ≤3months** |  | The mean swal-qol change - follow-up ≤3months in the intervention groups was **12.96 higher** (19.58 lower to 45.5 higher) |  | 167 (3 studies) | ⊕⊕⊕⊝ **moderate**^1^ |  |
| **Adverse events** | **Study population** | | **OR 1.58**  (0.73 to 3.38) | 721 (5 studies) | ⊕⊕⊕⊕ **high** |  |
|  | **31 per 1000** | **48 per 1000** (23 to 97) |  |  |  |  |
|  | **Moderate** | |  |  |  |  |
|  | **0 per 1000** | **0 per 1000** (0 to 0) |  |  |  |  |
| **effective rate** | **Study population** | | **OR 2.54**  (2.05 to 3.16) | 2077 (12 studies) | ⊕⊕⊕⊕ **high** |  |
|  | **624 per 1000** | **809 per 1000** (773 to 840) |  |  |  |  |
|  | **Moderate** | |  |  |  |  |
|  | **689 per 1000** | **849 per 1000** (820 to 875) |  |  |  |  |
| **effective rate - all effective rate** | **Study population** | | **OR 2.63**  (1.97 to 3.53) | 1151 (12 studies) | ⊕⊕⊕⊕ **high** |  |
|  | **633 per 1000** | **819 per 1000** (773 to 859) |  |  |  |  |
|  | **Moderate** | |  |  |  |  |
|  | **689 per 1000** | **854 per 1000** (814 to 887) |  |  |  |  |
| **effective rate - effective rate according to VFSS** | **Study population** | | **OR 2.16**  (1.35 to 3.46) | 364 (3 studies) | ⊕⊕⊕⊕ **high** |  |
|  | **503 per 1000** | **686 per 1000** (577 to 778) |  |  |  |  |
|  | **Moderate** | |  |  |  |  |
|  | **600 per 1000** | **764 per 1000** (669 to 838) |  |  |  |  |
| **effective rate - effective rate according to WST** | **Study population** | | **OR 2.72**  (1.77 to 4.19) | 562 (5 studies) | ⊕⊕⊕⊕ **high** |  |
|  | **700 per 1000** | **864 per 1000** (805 to 907) |  |  |  |  |
|  | **Moderate** | |  |  |  |  |
|  | **700 per 1000** | **864 per 1000** (805 to 907) |  |  |  |  |
| *The basis for the **assumed risk** (e.g. the median control group risk across studies) is provided in footnotes. The **corresponding risk** (and its 95% confidence interval) is based on the assumed risk in the comparison group and the **relative effect** of the intervention (and its 95% CI).  **CI:** Confidence interval; **OR:** Odds ratio; | | | | | | |
| GRADE Working Group grades of evidence **High quality:** Further research is very unlikely to change our confidence in the estimate of effect.  **Moderate quality:** Further research is likely to have an important impact on our confidence in the estimate of effect and may change the estimate. **Low quality:** Further research is very likely to have an important impact on our confidence in the estimate of effect and is likely to change the estimate. **Very low quality:** We are very uncertain about the estimate. | | | | | | |
| ^1^ The sample size is insufficient. ^2^ Total results are inconsistent with subgroup results. | | | | | | |
